# Quarterly injectable hormonal contraceptive does not increase the activity of the renin-angiotensin-aldosterone system in women without cardiovascular risk factors

**DOI:** 10.61622/rbgo/2025rbgo71

**Published:** 2025-11-18

**Authors:** Alice Miranda de Oliveira, Priscilla Araújo dos Santos, Pedro Elias Santos Souza, Marvyn de Santana do Sacramento, Clóvis Figueiredo Souza, Ana Marice Texeira Ladeia, Jefferson Petto

**Affiliations:** 1 Escola Bahiana de Medicina e Saúde Pública Salvador BA Brasil Escola Bahiana de Medicina e Saúde Pública, Salvador, BA, Brasil.; 2 Actus Cordios Reabilitação Cardiovascular, Respiratória e Metabólica Salvador BA Brasil Actus Cordios Reabilitação Cardiovascular, Respiratória e Metabólica, Salvador, BA, Brasil.; 3 Faculdade Atenas Valença BA Brasil Faculdade Atenas, Valença, BA, Brasil.; 4 Faculdade Zarns Salvador BA Brasil Faculdade Zarns, Salvador, BA, Brasil.

**Keywords:** Contraceptives agents, Hormonal contraceptives, Renin-angiotensin system, Aldosterone, Blood pressure, Women's health

## Abstract

**Objective:**

To test the hypothesis that the quarterly injectable contraceptive increases the activity of the renin-angiotensin-aldosterone system.

**Methods:**

This was a cross-sectional observational study that included eutrophic, irregularly active women, aged between 18 and 30 years, who had been taking quarterly injectable contraceptive (150mg of medroxyprogesterone acetate) for at least 6 months or who had not used any type of hormonal contraceptive for at least 6 months. At first the volunteers underwent a physical examination and answered a standard questionnaire. They were then sent for blood collection of laboratory variables: plasma renin activity and concentration, angiotensin-converting enzyme 1 (ACE 1), and aldosterone. The data was analyzed using the two-tailed Student's t-test, with significance <0.05.

**Results:**

Sixty-two women were included in this study, divided into the Injectable Contraceptive Group (ICG) with n=23 and the No Contraceptive Group (NCG) with n=39. ICG had lower mean plasma renin activity values than NCG, respectively 0.4 ± 0.17 vs 1 ± 0.6 (p <0.01). The mean values for plasma renin concentration, ACE 1, and aldosterone did not differ between the groups (respectively, p= 0.21; 0.66; 0.09).

**Conclusion:**

Women using quarterly injectable contraceptives do not show greater activity of the renin-angiotensin-aldosterone system than their counterparts who do not use this drug.

## Introduction

According to the 2019 National Health Survey, more than 80% of Brazilian women of reproductive age use some form of contraception.^(1)^ Although the most widely used method continues to be the combined oral contraceptive (COC), approximately 10% of this population uses the injectable contraceptive (CI).^(1)^ In Brazil, CI is a method provided by the Unified Health System (UHS) as a strategy to guarantee the right to family planning. It is considered a common option among women who prefer long-acting methods and with less need for frequent monitoring, such as the quarterly CI composed of medroxyprogesterone acetate (prevents contraception for an average of 10 months after the last injection).^(2)^

Since 2013 our research group has been studying the use of COCs in women without other risk factors. Among several aspects, we observed that these women present median values of plasma renin activity 5 times higher when compared to their counterparts without the use of hormonal contraceptives.^(3)^ In a recent systematic review, we concluded that the values of the renin substrate (angiotensinogen), plasma renin activity, angiotensin II and aldosterone are increased in women who use COCs.^(4)^ This review suggests that the main triggering factor for increased activity of the renin-angiotensin-aldosterone system (RAAS) is the presence of ethinylestradiol in the composition of COCs.^(4)^ This synthetic agent increases the production of hepatic angiotensinogen messenger RNA, promotes increased plasma renin activity and increases the activity of the main axis of this system.^(4,5)^ Other studies reinforce the risk of increased RAAS activity and blood pressure in COC users caused by ethinylestradiol, as shown in the systematic review that evaluated the potential effects of COC on blood pressure.^(6)^

However, studies conducted by Oelkers et al.,^(7,8)^ indicate that not only ethinylestradiol, but also oral progestin increases the production and activity of plasma renin and the concentration of aldosterone. This suggests that progestins also potentially increase the activity of the RAAS. In our literature searches, we found only one observational study that described the effect of quarterly injectable progestins on the RAAS.^(9)^ The results indicate that there is no change in the RAAS molecules in women using injectable progestins when compared to the group without contraceptives. When compared to the COC group, only aldosterone showed a significant difference, being higher in women using COC.^(9)^

However, we must consider that the aforementioned study was conducted with the African population and combined the intrauterine device (levonorgestrel), the subdermal implant (etonogestrel) and the quarterly injectable contraceptive (medroxyprogesterone acetate) in the same group. Furthermore, the study directly investigated only two substances of the RAAS, angiotensin II and aldosterone, using an indirect method through the values of angiotensin II to estimate renin activity.

Unlike what was observed in the literature,^(9)^ our study evaluated the RAAS through the activity and concentration of plasma renin, angiotensin-converting enzyme 1 (ACE 1) and aldosterone. Therefore, the objective of this study was to test the hypothesis that the quarterly injectable contraceptive, based on medroxyprogesterone acetate, increases the activity of this system.

## Methods

This was a cross-sectional observational study. The study included women aged between 18 and 30 years, who were eutrophic, irregularly active according to the International Physical Activity Questionnaire (IPAQ) - short version^(10)^ and who had been taking a quarterly CI consisting of medroxyprogesterone acetate for at least 6 months (two doses) or who had not been using any type of hormonal contraceptive for at least 6 months.

Women with a waist circumference above 88cm, hypertensive or taking antihypertensive drugs, with heart, kidney, and/or metabolic diseases, liver dysfunction, smokers and/or alcoholics assessed by the CAGE questionnaire were excluded. In addition, women with polycystic ovary syndrome due to complications of menstrual cycle deregulation, endometrial cancer, and women with elevated inflammatory markers compatible with infection, in this case ultrasensitive C-Reactive Protein above 10mg/L, were excluded.^(11)^

The sample was divided into two groups: 1) the Injectable Contraceptive Group (ICG) made up of women who had been using medroxyprogesterone acetate (150mg) every three months for at least six consecutive months, and 2) the No Contraceptive Group (NCG) made up of women who had not been using any hormonal contraceptive method for at least six consecutive months.

The outcome variables assessed were: plasma renin activity and concentration, Angiotensin-Converting Enzyme 1 (ACE 1), and aldosterone.

At first, all the volunteers underwent a physical examination and answered a standard questionnaire, both designed to gather general information about the characteristics of the sample. The physical examination consisted of measuring heart rate and blood pressure at rest, total body mass, and height and then calculating Body Mass Index (BMI) and waist circumference.

A Choicemmed wrist cardio-frequency meter was used to measure heart rate. To measure blood pressure, we followed the recommendations of the American Heart Association and used an adult sphygmomanometer duly calibrated by the National Metrology Institute (INMETRO) and a Premyum stethoscope.

Height was measured using a professional Sanny stadiometer with an accuracy of 0.1cm, carried out with the subjects barefoot and with their buttocks and shoulders resting on a vertical backrest. Total body mass was measured using a Filizola digital scale, with a maximum capacity of 150kg, calibrated by INMETRO and with its own certificate specifying a margin of error of ±100g.

BMI was calculated using mass and height measurements, according to the following formula: BMI = mass(kg) / height(m^2^). The BMI cut-off points adopted were those recommended by the World Health Organization,^(12)^ being underweight (BMI < 18.5); eutrophy (BMI 18.5-24.9); overweight (BMI 25-29.9) and obesity (BMI ≥ 30).

A Wiso flexible metal tape measure was used to measure waist circumference, with a measurement setting of 0.1cm. The measurement was performed at the smallest curvature located between the ribs and the iliac crest, without compression of the tissues. When it was not possible to identify the smallest curvature, the measurement was taken two centimeters above the umbilical scar. The cut-off points adopted for waist circumference were stipulated according to the degree of substantial risk for metabolic syndrome, with ≥ 88cm for women.^(13)^

In the second phase, all the volunteers were instructed to fast for 12 hours, not to change their diet during the week of the test, not to make any physical effort other than usual and not to drink alcohol 24 hours before the laboratory test. They were then sent to the laboratory to have their blood samples taken.

The volunteers were instructed to rest in the supine position for 30 minutes as soon as they arrived at the laboratory.^(14)^ After resting, the following laboratory variables were collected: Glutamic Oxalacetic Transaminase (GOT) and Glutamic Pyruvic Transaminase (GPT) to assess liver function, fasting glucose to assess blood glucose levels, c-reactive protein to assess inflammatory markers and plasma renin, ACE 1 and aldosterone concentrations to assess the RAAS.

**Figure 1:**
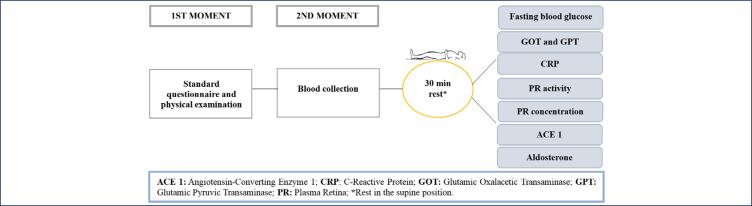
Data collection protocol

The values for GOT and GPT were obtained using the UV kinetic method, the values for fasting glucose using the enzymatic colorimetric method, and the values for ultrasensitive c-reactive protein using the turbidimetry method. Plasma renin concentration was measured by the chemiluminescence method, ACE 1 values were obtained by the kinetic spectrophotometry method and aldosterone by the chemiluminescence method. The organization of the collections can be seen in figure 1.

Initially, symmetry and kurtosis tests and the Shapiro-Wilk test were applied to check the distribution of the data. As there was no unequal variance between the samples, all the variables were described as mean and standard deviation. The two-way unpaired Student's t-test was used to compare the variables between groups. The significance level adopted for this study was 5% and all the data was analyzed using the BioEstat for Windows version 5.0 statistical software.

Sample sufficiency was calculated using plasma renin concentration values as a reference. A pilot study was carried out with ten women, five in each group, in which the mean and standard deviation of plasma renin concentration were, respectively, 20 ± 8.95 for ICG and 9 ± 7.14 for NCG. Based on these data, the sample was calculated using the BioEstat 5.0 for Windows statistical program, with an alpha of 0.05 and a beta of 0.8, which resulted in a sample size of 8 women in each group. However, considering that the laboratory coefficient of variation of plasma renin dosage is 3% and that a difference 3 times greater than expected cancels out the bias of this analytical coefficient of variation, 44 women were then needed to compose the study sample, with 22 volunteers in each group.

This study was approved by the Ethics Committee of the Bahiana School of Medicine and Public Health under *Certificado de Apresentação para Apreciação Ética* – CAAE No. 35292220.2.0000.5544 and Ethics Committee Opinion No. 4461049. All volunteers were previously informed about the study and signed an Informed Consent Form.

## Results

Sixty-two women were included in this study, 23 of whom were in the IC group (ICG). The clinical and anthropometric characteristics of the sample are described in table 1. waist circumference and GOT are the only variables that differed between the groups, being higher in the group using IC (p< 0.01 and 0.05 respectively). The other variables were homogeneous between the groups.

**Table 1:** Clinical and anthropometric characteristics of the sample

Variables	ICG (n = 23)	NCG (n = 39)	p- value*	Normality value
Age (years)	23 ± 3.2	23 ± 3.3	0.94	-
Body mass index (kg/m^2^)	23 ± 3.7	21 ± 1.9	0.13	18.5 a 24.9
Waist circumference (cm)	78 ± 6.1	73 ± 5.5	<0.01	< 88 cm
Resting heart rate (bpm)	82 ± 9.5	78 ± 8.6	0.16	50 a 100
Systolic blood pressure (mmHg)	110 ± 9.1	107 ± 9.6	0.17	Up to 139
Diastolic blood pressure (mmHg)	74 ± 7.6	72 ± 9.1	0.55	Up to 89
Fasting blood glucose (mg/dL)	81 ± 8.6	82 ± 6.2	0.68	< 100
Glutamic oxaloacetic transaminase (U/L)	22 ± 9.0	17 ± 5.6	0.05	Up to 35
Glutamic pyruvic transaminase (U/L)	22 ± 14.6	16 ± 9.3	0.15	Up to 35
C-reactive protein (mg/L)	1.3 ± 0.8	1.0 ± 0.8	0.45	Up to 1
Duration of IC use (months)	32 ± 34.9	-	-	-

IC: Injectable Contraceptive; ICG: Injectable Contraceptive Group; NCG: No Contraceptive Group;

* Student's t-test

Table 2 shows the values for plasma renin activity, plasma renin concentration, ACE 1, and aldosterone for both groups. It is noteworthy that there was a difference between the groups for plasma renin activity, with NCG showing higher mean values than ICG (p< 0.01), with no difference for the other variables.

**Table 2:** Plasma renin concentration values, angiotensin-converting enzyme-1, and aldosterone concentration values of the two groups

Variables	ICG (n = 23)	NCG (n = 39)	95% CI	p-value*	Normality value
Plasma renin activity (ng/mL/H)	0.4 ± 0.17	1 ± 0.6	-0.87 a - 0.30	<0.01	0,32 a 1,84
Plasma renin concentration (uUI/mL)	18 ± 11.8	13 ± 8.7	-2.90 a 12.49	0.21	2.8 a 39.9
Angiotensin-1 converting enzyme (U/L)	33 ± 12.7	34 ± 13.8	-9.41 a 6.05	0.66	9 a 67
Aldosterone (ng/dL)	8 ± 2.8	10 ± 3.8	-3.87 a 0.19	0.09	1.8 a 23.2

CI: Confidence Interval; ICG: Injectable Contraceptive Group; NCG: No Contraceptive Group;

* Student's t-test

## Discussion

The results of this study indicate that the values of the substances that make up the RAAS axis in women who use IC and do not use contraceptives are within the normal range. However, although the concentration of plasma renin, ACE 1, and aldosterone did not differ between the groups, plasma renin activity was lower in the group using IC.

Plasma renin is the enzyme responsible for initiating the RAAS. Released by the cells of the renal juxtaglomerular apparatus, its function is to cleave angiotensinogen (commonly known as the plasma substrate for renin) to form angiotensin I.^(15)^ Renin can be assessed both by its plasma concentration and by its activity. In the literature review produced by our group,^(4)^ we pointed out that the use of COC increases both the concentration and activity of plasma renin, a different result to that obtained in this study. This implies that unlike COC, which is composed of ethinylestradiol and progestin, IC, which is composed only of progestin and administered quarterly, does not trigger this increase in RAAS activity.

This difference in results between the use of IC and COC can be explained by subclinical inflammation. Studies carried out with women of reproductive age using COC showed higher CRP values.^(3,16)^ The unfavorable inflammatory profile justifies both the higher activity and the higher concentration of plasma renin in this population since subclinical inflammation feeds back into renin production.^(15)^ When we analyzed the ICG CRP values described in Table 1, we observed that there was no difference in the inflammatory profile between the groups in this study. Therefore, it is suggested that there is no RAAS feedback caused by subclinical inflammation in women taking quarterly IC.

In addition, our study provides another apparent counterpoint. Unlike the studies by Oelkers et al.^(7,8)^ in which both renin activity and plasma aldosterone concentration were higher during progestin use, our study shows that plasma renin activity is lower in the group using quarterly IC. Therefore, IC does not seem to negatively influence renin production and activity, which consequently does not promote greater RAAS activity. This idea is confirmed when we look at an important final element of the RAAS, aldosterone, which, as we said, did not differ between the groups. A very reasonable hypothesis could explain this controversy. In the studies by Oelkers et al.^(7,8)^ the progestin investigated was drospirenone (it has antimineralocorticoid properties), while in our study the progestin evaluated was medroxyprogesterone acetate (it does not have antimineralocorticoid activity). At this point, antimineralocorticoid activity seems to play an important role, since the increase found in plasma renin activity and plasma aldosterone in the studies by Oelkers et al.^(7,8)^ could be interpreted as an endogenous counter-regulation to the antimineralocorticoid activity of drospirenone.

When comparing our results with the study developed by van Rooyen et al.^(9)^ we noticed that aldosterone presented a similar behavior in both studies, with no significant difference between the groups using IC and the group without contraceptives. However, the results of the variable plasma renin activity differed in the studies. Our study showed lower values of plasma renin activity in the IC group compared to women without contraceptives, while van Rooyen et al found no difference between the groups. This difference between the studies can be justified by the laboratory measurement method, since in the study by van Rooyen et al.^(9)^ plasma renin activity was estimated through angiotensin II values.

It is interesting to note that waist circumference and GOT values were higher in ICG. Increased waist circumference is an important predictor of subclinical inflammation,^(17)^ an unfavorable lipid profile,^(18)^ and greater cardiometabolic risk.^(19,20)^ As for GOT, we know that higher values can be associated with hepatic, renal, or cardiac alterations.^(21)^ The literature shows that, via the parenteral route, the liver is still the main source of progestin metabolism, although it receives lower concentrations of metabolites because there is no first hepatic passage as in the oral route.^(22)^ Taking into account the influence of the route of administration, it seems to us that the use of injectable progestins may have led to higher GOT levels in the ICG. Despite all this, we cannot disregard the fact that all values are within normal ranges, although the differences found reinforce the need for medium and long-term monitoring of the cardiometabolic profile of women using quarterly IC.

However, this study suggests that plasma renin activity is lower in women using IC and that this method does not promote an increase in RAAS activity, contrary to what was presented in previous studies with women using COC,^(3,4)^ taking into account the differences already mentioned. In line with this idea, systematic reviews show that hormonal contraceptives composed only of progestins are safer and present a lower risk of pulmonary thromboembolism in women with sickle cell disease, for example.^(23,24)^

Finally, in light of what has been presented and discussed here, in the long term, we can put forward the hypothesis that quarterly IC does not have a negative impact on RAAS, refuting the idea that this type of contraceptive could favor the development of systemic arterial hypertension. However, this and other hypotheses put forward by our research should be tested and confirmed by longitudinal studies with larger samples that assess cause-and-effect relationships.

## Conclusion

This study suggests that women using quarterly injectable hormonal contraceptives do not show a higher activity of the renin-angiotensin-aldosterone system than their counterparts who do not use this drug. Furthermore, it has been shown that plasma renin activity is lower in women taking quarterly injectable hormonal contraceptives. These data may suggest that injectable hormonal contraceptives do not have an unfavorable risk profile in terms of changes in RAAS and should be evaluated in clinical trials as a possible contraceptive option for hypertensive women.
